# A case misdiagnosed as hyperthyroid heart disease: Surgical repair of unroofed coronary sinus syndrome with severe tricuspid regurgitation

**DOI:** 10.1002/ccr3.7990

**Published:** 2023-09-28

**Authors:** Xuejiao Ding, Zhimin Liao, Yichao Tao, Xiaofeng Wu

**Affiliations:** ^1^ Department of Ultrasound Imaging, Xiaogan Hospital Affiliated to Wuhan University of Science and Technology, Xiaogan Central Hospital Xiaogan China; ^2^ Department of Cardiothoracic Surgery Xiaogan Hospital Affiliated to Wuhan University of Science and Technology, Xiaogan Central Hospital Xiaogan China

**Keywords:** echocardiography, heart disease, surgery, unroofed coronary sinus syndrome (URCS)

## Abstract

Unroofed coronary sinus syndrome (URCS), also known as coronary sinus septal defect, is a rare congenital heart disease. Because of its special anatomy and the lack of typical clinical symptoms, the disease is easily missed and misdiagnosed. This case report particularly describes a middle‐aged male patient with URCS misdiagnosed for more than 19 years, covered by hyperthyroid heart disease, who subsequently developed uncontrollable symptoms such as chest tightness and shortness of breath and came to our hospital. After a clear diagnosis in our hospital, the patient was successfully cured after treatment with coronary sinus repair and tricuspid valvuloplasty under extracorporeal circulation.

## Graphical abstract

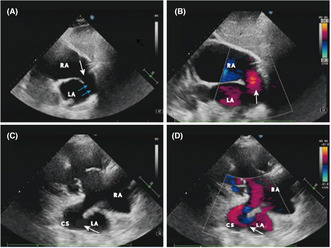

## 1 INTRODUCTION

Unroofed coronary sinus syndrome (URCS) is a rare congenital anomaly of heart disease first reported by Raghib et al. in 1965.^1^ During embryonic life, the top of the coronary sinus(CS) and its corresponding posterior wall of the left atrium (LA) are partially or completely defective due to incomplete formation of the left atrial venous folds, resulting in direct communication between the CS and the LA.^2^, ^3^, ^4^ The size of the defect and the degree of left‐to‐right shunt usually determine the clinical presentation, which is nonspecific.^5^, ^6^ This disease is very easy to be missed and misdiagnosed in the clinic, especially in isolated anaplastic coronary sinus syndrome.^7^ Here, we report a 54‐year‐old male patient with URCS who had been misdiagnosed for more than 19 years due to a combination of hyperthyroid heart disease, a case of misdiagnosis that has not been reported. We successfully repaired the defect in the CS through surgical treatment.

## 2 CASE REPORT

**FIGURE 1:**
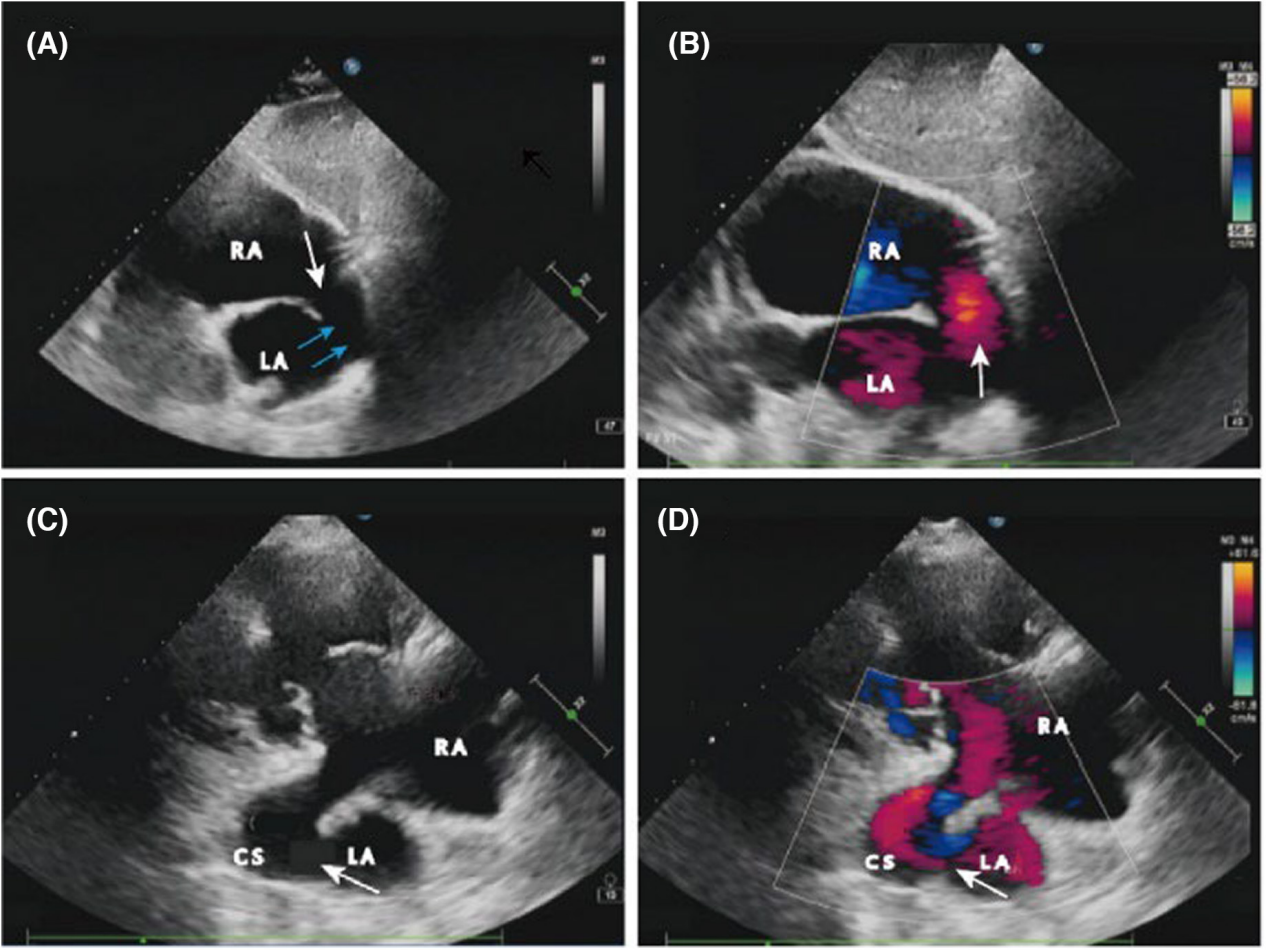
Preoperative two‐dimensional echocardiography. (A, B). Subxiphoid two‐atrial view: (A). The two‐dimensional echocardiogram shows the total absence of the sinus wall between the LA and CS and the interrupted echo of the posterior lower part of the atrial septum (blue arrows show the sinus wall, white arrows show the site of the septal defect); (B). Color Doppler shows the direct flow of blood from the LA into RA through the coronary venous sinus defect and the atrial septal defect (white arrows show); (C, D). Section of the right ventricular inflow view: (C). Two‐dimensional ultrasound shows dilated coronary sinus and coronary sinus wall defect (shown by white arrows); (D) color Doppler shows blood flow from the LA directly into RA through the CS sinus wall defect (shown by white arrows). (LA, left atrium; RA, right atrium; CS, coronary sinus).

A 54‐year‐old male patient was diagnosed with hyperthyroid heart disease 19 years ago due to palpitations, tremors, fear of heat, hunger, shortness of breath, chest tightness, and other symptoms in another hospital, and then took methimazole 20 mg/day or 10 mg/day intermittently. The patient felt chest tightness and shortness of breath 2 months ago, and now he came to our hospital with uncontrollable symptoms. Physical examination showed that blood pressure was 136/98 mmHg, pulse was 77 beats/min, respiration was 18 beats/min, heart rhythm was irregular, and the first heart sound was variable in intensity. After admission, he was admitted to the endocrinology department of our hospital because of his previous diagnosis of hyperthyroid heart disease. Thyroid function examination showed that free triiodothyronine (FT3) was 8.7 pmol/L, free thyroxine (FT4) was 27 pmol/L, thyroid stimulating hormone (TSH) was 0.15 uIU/ml, and thyrotropin receptor antibody was 4.14 IU/L. The first transthoracic echocardiogram showed an enlarged right heart, severe tricuspid regurgitation, moderate pulmonary hypertension, and an irregular heart rate. The patient was considered to be in need of severe valve repair and was referred to cardiothoracic surgery for elective surgery. After preoperative anticoagulation, antihypertensive, and heart rate control treatment, a routine preoperative transthoracic echocardiogram was performed, and surprisingly, the patient's dilated CS with a diameter of about 2.07 cm could be seen in the subxiphoid biventricular view and the right ventricular inflow tract view. Dynamic observation shows that the CS is in direct communication with the LA, and there is a common septal defect between the CS and the LA (Figure 1A,C). Color Doppler showed a significant left‐to‐right shunt between the CS and the LA (Figure 1B,D), and a large amount of regurgitant signal was seen on the right atrial (RA) side of the tricuspid orifice during systole (Figure S1A), with a regurgitant velocity of about 3 m/s and a regurgitant pressure difference of 36 mmHg, and a pulmonary artery systolic pressure of 51 mmHg estimated by the tricuspid regurgitant pressure difference.

**FIGURE 2:**
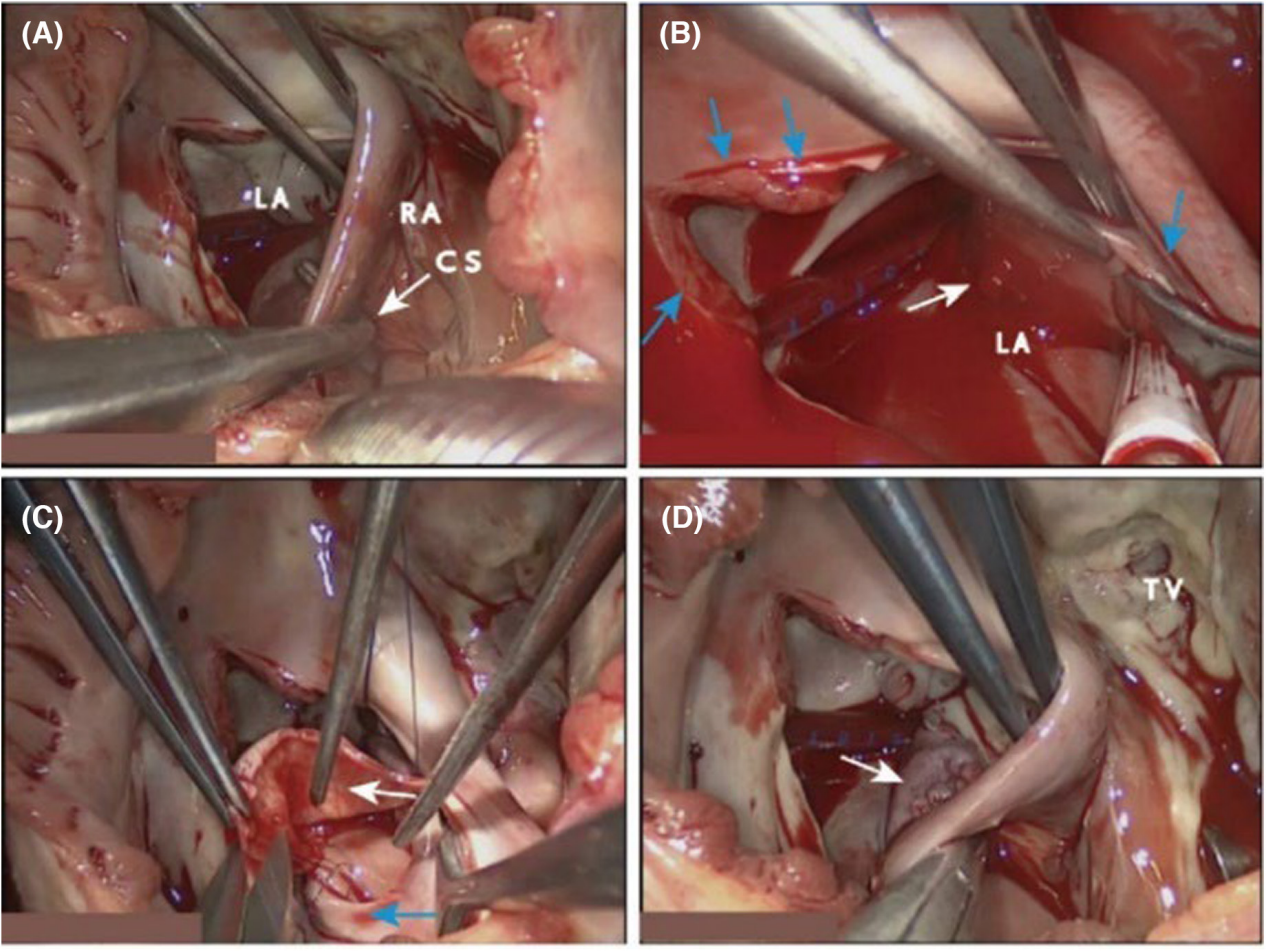
Intraoperative pictures. (A). Surgical exploration reveals an atrial septal defect of the coronary sinus type (White arrow shows right‐angle forceps probe into the LA from the opening of coronary sinus of the RA); (B). When the aortic root is perfused with cardiac arrest fluid, the left coronary vein is seen to open directly into the LA (white arrow indicates where blood flow is visible; blue arrow shows the atrial septum); (C). Reconstruction of the coronary vein and repair of the atrial septal defect with autologous pericardium, and repair of the defect is completed by suturing the right edge of the autologous pericardial patch (white arrow shows the autologous pericardium; the blue arrow shows the edge of the atrial septal defect); (D). After repair, the left coronary vein was introduced into RA, and the atrial septal defect repair was completed at the same time (white arrow shows). (LA, left atrium; RA, right atrium; CS, coronary sinus; TV, tricuspid valve).

**FIGURE 3:**
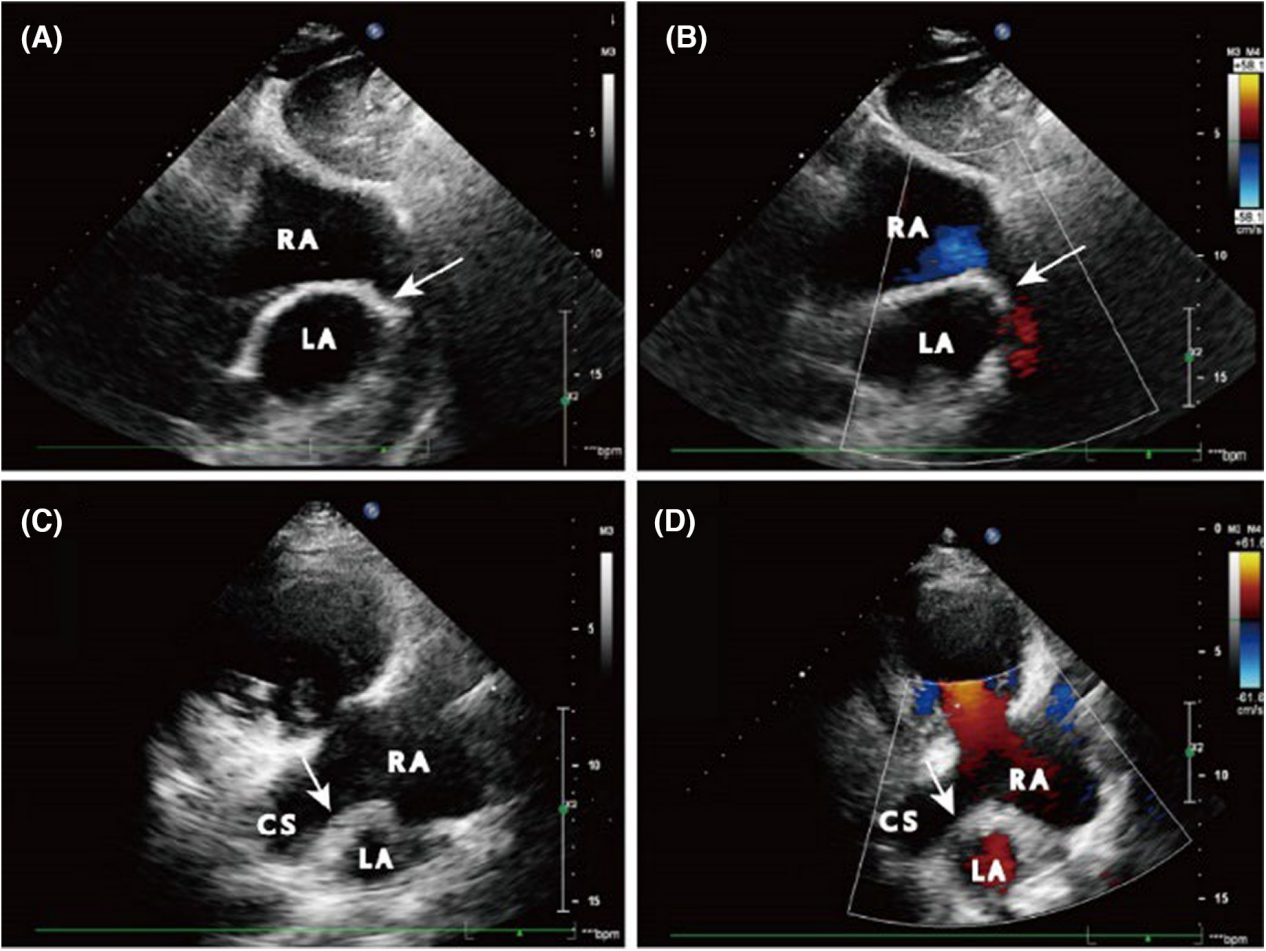
Two‐dimensional echocardiography at 1 week postoperatively. (A, B). Subxiphoid two‐atrial view: (A). two‐dimensional ultrasound shows no significant interruption between the LA and CS as well as the atrial septum (shown by white arrows); (B). Color Doppler shows no color shunt signal between the LA and CS (shown by white arrows); (C, D). Long‐axis view of the left ventricle: (C). Two‐dimensional ultrasound shows an obvious interval echo between LA and CS (shown by the white arrow); (D). Color Doppler shows that there is no blood flow signal connecting the two (shown by white arrows). (LA, left atrium; RA, right atrium; RV, right ventricle; CS, coronary venous sinus).

After a series of examinations and clinical observations, the patient was finally diagnosed with congenital heart disease, URCS with severe tricuspid regurgitation. During hospitalization and symptomatic supportive treatment such as control of hyperthyroidism, hypotension, anticoagulation, and ventricular rate control, the patient was determined to have a clear indication for surgery after a comprehensive evaluation by our thoracic surgery department and therefore underwent coronary sinus repair and tricuspid valvuloplasty under extracorporeal circulation in our hospital. During surgery, a coronary venous sinus‐type atrial septal defect was seen (Figure 2A), measuring approximately 30 × 30 mm, with a significantly enlarged tricuspid annulus and a CS opening directly into the LA (Figure 2B), which was consistent with the echocardiographic findings. The left coronary vein tunnel was created with an autologous pericardial patch, and the right side of the patch was sutured to the contiguous edge of the atrial septal defect (Figure 2C), and the left coronary vein was drained into the RA and the atrial septal defect was repaired (Figure 2D). Close the atrial septal gap, cut open the edge of the right atrial incision, fully expose the tricuspid valve, measure the size of the tricuspid valve ring, and insert a 28 mm Edward hard Carpentier shaped ring, using 6 × 14 double headed needles were intermittently sutured along the anterior and posterior annulus of the tricuspid valve in a mattress style with 11 needles, and there was no significant regurgitation of the tricuspid valve during water testing. The patient's symptoms of chest distress and asthma improved significantly 1 week after the surgery, and Echocardiographic review showed that there was no echogenic interruption between the CS and the LA (Figure 3A,C), and there was no clear residual shunt signal between them (Figure 3B,D), and only a trace regurgitant signal was seen in the tricuspid orifice during systole (Figure S1B). The patient's condition was stable after surgery, and he was discharged from the hospital 14 days later.

## 3 DISCUSSION

URCS is a congenital abnormal developmental heart disease with an incidence of only 0.2%–0.3%, which is an extremely rare heart disease. It is a type of atrial septal defect and accounts for less than 1% of all atrial septal defects.^8^, ^9^ Because the CS is partially or completely unroofed, the blood flow between CS and LA can be interconnected.^10^ When the CS is connected to the LA, the blood flow in the RA does not go through the oxygen exchange of the pulmonary capillaries and flows directly into the LA, resulting in the connection between the RA and the LA, which will lead to a left‐to‐right or right‐to‐left shunt, and the blood flowing into the RA will increase the preload of the right heart, thus increasing the size of the right heart, which can lead to pulmonary hypertension and right heart failure in serious cases.^11^ When unoxygenated venous blood flows directly into the LA, patients may experience chest tightness, chest pain, shortness of breath, hypoxemia, and, in some cases, atrial fibrillation and, in a few cases, brain abscess and cerebral embolism.^12^ However, the clinical symptoms are generally nonspecific. Although there are reports in the literature about the URCS, there are very few reported cases of misdiagnosis and underdiagnosis of the URCS, especially those misdiagnosed as hyperthyroidism heart disease. Here, we report a case of URCS combined with hyperthyroid syndrome, misdiagnosed as hyperthyroid heart disease.

In the case we reported, URCS is isolated, which is uncommon. This patient also had hyperthyroidism on top of URCS, and a large amount of thyroid hormone acts on the heart for a long time, which enhances myocardial metabolism and increases oxygen consumption. Long‐term overload can enlarge the right heart, and the enlarged right heart causes severe tricuspid regurgitation, while atrial fibrillation is the most frequent tachyarrhythmia in hyperthyroid patients. Both can cause right heart enlargement, pulmonary hypertension, and tricuspid regurgitation as well as atrial fibrillation, so the right heart enlargement combined with tricuspid regurgitation caused by a left‐to‐right shunt in the URCS was misdiagnosed as a result of hyperthyroidism. In addition, because the patient showed typical signs and symptoms of hyperthyroid heart disease, such as panic, shaking hands, chest tightness, and asthma, while the clinical symptoms caused by the URCS syndrome were not specific, the patient had been overlooked for more than 10 years and at the time of the first echocardiogram at our institution.

Related studies have reported that before the era of echocardiography, accurate diagnosis of URCS could only be achieved during surgical procedures or autopsy.^13^, ^14^, ^15^ Transthoracic echocardiography is the preferred method for the diagnosis of this disease;^7^ however, the septal defect of the unroofed coronary sinus is located in the posterior lower part of the atrial septum, and the defect can barely be explored in the conventional view of echocardiography. In addition, because the CS is located posterior to the heart and transthoracic echocardiography is subject to the patient's acoustic window and position as well as the examining physician's technique, it is also easily missed and misdiagnosed. As in this case report, the results of the two transthoracic echocardiograms are different due to the different physicians, so transthoracic echocardiography has some limitations in the URCS, and the diagnosis of the disease is not completely confirmed. Related studies have reported that the diagnostic accuracy of echocardiography for URCS is only 65%, and not all URCS can be diagnosed preoperatively.^16^, ^17^ Surgical treatment for secondary tricuspid regurgitation associated with the tricuspid valve often includes the Kay method, the De Vega method, and the Carpentier‐Edwards (C‐E) rigid ring. Among them, the Carpentier‐Edwards (C‐E) rigid ring has been widely accepted for tricuspid annuloplasty.^18^, ^19^ In the case reported in this article, we used the last method.

In summary, URCS is easily missed and misdiagnosed because of its structural peculiarities and nonspecific clinical manifestations, especially when combined with other diseases that cause cardiac changes, such as URCS combined with hyperthyroid heart disease in this case report. Echocardiography is the preferred diagnostic method for this disease, but it cannot be fully diagnosed due to some objective and subjective factors, so repeated dynamic observation of multiple views of the heart must be performed when the disease is suspected, and if necessary, a combined real‐time three‐dimensional transesophageal echocardiography can be performed to improve the diagnosis rate and give treatment as early as possible. In the meantime, URCS requires surgical repair. At our institution, coronary sinus repair under transthoracic extracorporeal circulation is the recommended surgical procedure for URCS.

## AUTHOR CONTRIBUTIONS

**Xuejiao Ding:** Writing – original draft. **Zhimin Liao:** Resources. **Yichao Tao:** Software; supervision. **Xiaofeng Wu:** Writing – review and editing.

## FUNDING INFORMATION

None.

## CONFLICT OF INTEREST STATEMENT

The authors declare that there are no conflicts of interest.

## CONSENT

The authors confirm that written consent has been obtained from the patient for submission and publication. A copy of the patient's consent for publication is available for review by the editor of the journal.

## ETHICS STATEMENT

This study was approved by the Ethics Committee of Xiaogan Hospital Affiliated to Wuhan University of Science and Technology. The research involved one patient and their tissues, and informed consent for the use of their clinical images and tissues was obtained from the patient.

## Supporting information


Figure S1.

## Data Availability

The data that support the findings of this study are available from the corresponding author upon reasonable request.
