# Supplementary material for: A case misdiagnosed as hyperthyroid heart disease: Surgical repair of unroofed coronary sinus syndrome with severe tricuspid regurgitation
Source: Clin Case Rep. 2023 Sep 28;11(10):e7990. doi: 10.1002/ccr3.7990 (PMC10539682; doi:10.1002/ccr3.7990)
Supplement: Supplementary file 1 — Figure S1. [file CCR3-11-e7990-s001.docx]

**Supplementary**


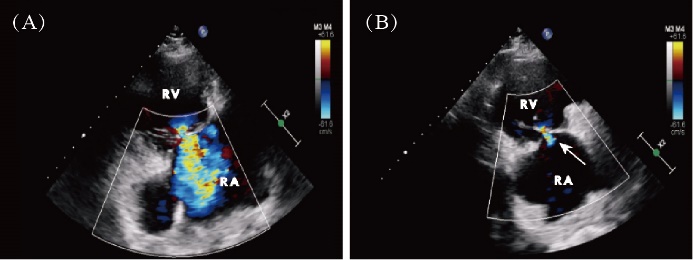


**Figure1.Comparative echocardiograms before and after tricuspid valve surgery.** (A). The preoperative two-dimensional echocardiogram shows a large amount of blue regurgitant signal on the RA side of the tricuspid orifice during systole, (B). The postoperative echocardiogram shows a small amount of regurgitant signal on the RA side of the tricuspid orifice during tricuspid valve closure (shown by the white arrow). (RA: right atrium; RV: right ventricle).
